# Psychopathological profile and quality of life of patients with oral lichen planus

**DOI:** 10.1590/1678-7757-2017-0146

**Published:** 2018-01-16

**Authors:** Małgorzata Radwan-Oczko, Edyta Zwyrtek, Joanna Elżbieta Owczarek, Dorota Szcześniak

**Affiliations:** 1 Wroclaw Medical University, Department of Oral Pathology, Wroclaw, Poland; 2 Lower Silesian Mental Health Center, Wroclaw, Poland; 3 Wroclaw Medical University, Department of Psychiatry, Division of Consultation Psychiatry and Neuroscience, Wroclaw, Poland

**Keywords:** Oral lichen planus, Psychopathology, Mental status

## Abstract

**Objectives:**

Oral lichen planus (OLP) is a chronic, multifocal, sometimes painful, inflammatory disease of the oral mucosa. OLP can predispose development of psycho-emotional disorders. Until now, the relationship between the severity of lichen planus and the psychological profile of patients (psychological well-being, perceived stress and pain coping strategies) has never been studied.

**Material and Methods:**

Study was conducted on 42 OLP patients. Number of sites involved, severity and activity score of OLP were evaluated. Psychological tests were used to evaluate patients’ psycho-emotional condition. The mean duration time of symptomatic OLP was 43 months.

**Results:**

We detected that the longer the duration of subjective symptoms, the poorer the quality of life and the higher the level of perceived stress (PSS). Also, the higher the PSS results, the greater the anxiety and depression on Hospital Anxiety and Depression Scale (HADS). Likewise, higher level of depression in HADS was strongly correlated with worse quality of life. (p≤0.05).

**Conclusions:**

In this study, we detected a relationship between duration of the disease, level of perceived stress and quality of life. The longer the disease lasts, the higher it tends to catastrophize. This may influence development or increase of the anxiety and depression and may decrease patients’ quality of life.

## Introduction

Oral lichen planus (OLP) is a well-known and common chronic inflammatory disease of the oral mucosa. Years of broad clinical, histological, immunological, immunohistochemical and genetic investigations have not identified the initial trigger and pathogenic pathway that leads to the formation of lesions. Some general diseases, such as hypertension, diabetes, chronic liver disease and genetic predisposition have been listed as associated with the enigmatic etiology of the disease^1^ ^,^ ^18^.

The diagnostic criteria of oral lichen planus – which have been first defined and subsequently revised by the World Health Organization – are precise, well known and describe both clinical and histopathological features^17^.

Oral lichen planus lesions can be present throughout the oral cavity and may persist for a long time. Adults and women are more often affected than children and men. The lesions are typically numerous, symmetrical, and bilateral. The most common sites of lesions are the buccal mucosa, lateral borders of the tongue and the gingiva. Clinically, OLP occurs in the form of reticular, papular, plaque-like and erosive or atrophic and bullous lesions^15^. Reticular, papular and plaque-like forms are white keratotic lesions that form asymptomatic lesions and are incidentally discovered by the patient or during an intraoral clinical examination. However, some patients may feel oedema or roughness of the oral mucosa. The reticular form can transform into more advanced erosive, atrophic or bullous form. These occur in the form of red lesions and are associated with a burning sensation and/or pain triggered or intensified by spicy, hot or salty food products or other local, mechanical or chemical irritating factors. The signs and symptoms of OLP are typical and fluctuate over time. Painful lesions may impede eating, speaking and oral hygiene. OLP can predispose or even lead to the development of tiredness, anxiety, stress and cancerophobia, which can be based on the hypothesis of the premalignant nature of this disease^5^ ^,^ ^23^. This can have an impact on the patients’ everyday life and their attitude towards the disease and the possibility of recovery. Also, side effects of the treatment and lack of evident long-time improvement may worsen the psycho-emotional condition of the patients. Additionally, patients with OLP may have extra-oral manifestations of the disease on the skin, nails and genital mucosa^16^ ^,^ ^18^.

Currently, oral lichen planus is defined as a T-cell mediated inflammatory reaction based on various hypothetic immunopathogeneses related with antigen-specific and non-specific mechanisms, an autoimmune response and humoral immunity. However, the specific antigens evoking the immune response have not been identified to date^1^ ^,^ ^18^. The psychological profile of patients with OLP has been investigated, and the occurrence of stress and anxiety frequently correlated with an exacerbation of lichen planus oral lesions^5^. Thus, it was also postulated that OLP could be understood as a psychosomatic disorder^16^. Soto, et al.^20^ (2003) suggested a significant association between the level of stress and OLP. The level of anxiety was also higher in OLP patients in comparison with the control group. A further study, focused on psychopathological symptoms, demonstrated that the mean scores of hypochondriasis, depression and hysteria were significantly higher in subjects with OLP than in controls^8^. Furthermore, considering an inverse relation, Suresh, et al.^21^ (2015) investigated a group of patients with anxiety and observed a statistically significant higher prevalence of oral lichen planus in that group than in the control group.

The evaluation of severity and extent of the signs and symptoms of oral lichen planus is important and should be determined during each clinical examination. The evaluation should include the initial state and the development of the disease and the possible patients’ response to treatment.

There are no available reports on the relationship between the severity and signs of lichen planus and the psychological profile of patients in terms of the multi-aspects of psychological well-being related to perceived stress, pain coping strategies and psychopathological symptoms.

## Material and methods

### Study sample and procedure

The study was conducted on 42 patients with clinically and histologically confirmed oral lichen planus (OLP), who were treated in the Department of Periodontology, Division of Oral Pathology, of Wroclaw Medical University from 2012-2014. The approval for the research from Wroclaw Medical University Bioethical Commission was obtained. Inclusion criteria were: patients with diagnosis and history of oral lichen planus, without significant systemic diseases like: history of malignant diseases, hepatitis C infection and diagnosed psychiatric disorders. There was no dysplasia in histologically OLP tissues tested.

The form of OLP lesions, the sites involved, the type of lichen planus lesion, the severity and the activity were assessed in the clinical examination according to a modified scoring methodology by Escudier, et al.^6^ (2007). Only the involved sites were included, without soft palate and oropharynx region. The severity score of OLP lesion from 0 to 3 describes: only keratosis, keratosis with mild erythema, keratosis with marked erythema, presence of ulceration. The activity score defines the severity of each site. The maximum severity score possible for evaluated sites was 20 points with the use of this modified method of evaluation of lichen planus lesions. The maximum activity score was 60 points.

Patients’ age, duration of the disease – understood as the time from beginning of symptoms or diagnosis of the disease until the day of clinical examination with exacerbations and remissions –, presence of extra-oral skin manifestations and other mucosal and/or nail lichen planus lesions, as well as coexistent general disorders, were recorded. Subsequently, the psychological instruments listed below were administered.

### Instruments

The following instruments were used to assess the different aspects of the patients’ functionality:

– Visual Analogue Scale (VAS) to assess the severity of pain and/or discomfort in the oral cavity using the self-assessed Visual Analogue Scale (VAS)^2^ ^,^ ^12^.
– The Pain Coping Strategies Questionnaire [CSQ; Polish adaptation by Juczyński (2009)]^10^ ^,^ ^19^ was used to evaluate the different pain coping strategies. The questionnaire includes 42 statements and 2 questions that were scored from 0 to 6 (0 – never use it, 6 – always) points. Each statement is attributed to six cognitive and one behavioral pain coping strategy, including diverting attention, reinterpreting the pain sensation, using coping self-statements, ignoring sensations, praying and hoping, catastrophizing and increased behavioral activities. Each strategy can be scored from 0 to 36 and the higher score is related to a more frequent use of the strategy. The questions measure the effectiveness of pain control and ability to decrease pain. Both questions are scored from 0 to 6. Higher scores are attributed to greater ability to decrease and control pain.
– The Hospital Anxiety and Depression Scale [HADS; Polish adaptation by De Walden, et al.^14^ (2000)]^14^ ^,^ ^24^ is a brief 14-item, self-report assessment of anxiety and depression widely used in clinical practice. The scale is divided into an anxiety subscale (HADS-A) and a depression subscale (HADS-D), both containing 7 items. Each item can be scored from 0 to 3. Hence, the possible scores for the depression and anxiety subscale vary from 0 to 21 points. A result of 0 – 7 points for either subscale signifies a normal range, a result of 8 −10 points indicates a moderate risk of a mood disorder and 11 – 21 points indicate the probable presence of a mood disorder.
– The Perceived Stress Scale [PSS-10; Polish adaptation by Juczyński and Oglińska-Bulik^11^ (2009)]^3^ ^,^ ^10^ ^,^ ^11^ contains 10 items measuring the perception of stress. Each item can be scored from 0 to 4 points (0 – never, 4 – very often) and the total score varies from 0 to 40 points. Additionally, the results are divided into ten scores that are on a scale of 1 to 10. The scale assesses the level of stress caused by unpredictable, uncontrollable occurrences and the effectivity of coping strategies used during the last month. Higher scores indicate a greater level of perceived stress.
– The Psychological General Well-Being Index [PGWBI; Polish adaptation by Klocek and Kawecka-Jaszcz^13^ ^,^ ^15^ (2003)]^4^ ^,^ ^13^ ^,^ ^15^ assesses the quality of life and general well-being. Each of the 22 items can be scored from 0 to 5, and the global score varies from 0 (poor quality of life) to 110 points (good quality of life). Furthermore, each item is attributed to one of 6 dimensions including anxiety, depressed mood, positive well-being, self-control, general health and vitality. Higher scores indicate better quality of life in each dimension.

### Statistical analyses

The results were statistically analyzed. The number of cases (N), the mean (X), the median (M), the range (min-max), the upper and lower quartiles (25Q-75Q) and the standard deviation (SD) were calculated for all the obtained parameters. A correlation analysis was performed on chosen parameters by calculating the Pearson product-moment correlation coefficient (Pearson's r) or the Spearman's rank correlation coefficient. P=0.05 was considered statistically significant, while 0.05<p<0.1 was considered to indicate a trend. Statistical analysis was performed using the EPIINFO 7.1.1.14 (Atlanta, Georgia, USA) statistical software package (dated 2-07-2013).

## Results

### Study group characteristics

The patients in the study group were from 24 to 85 years old. The mean age was 59.6 (±12.44) years. There were 34 women (80.9%) and 8 men (19.1%) patients. All subjects were classified into categories depending on the presence of general comorbid diseases: "no diseases", "1 or 2 diseases", "3 or more diseases". Twelve subjects (28.6%) were found to have "no diseases", 19 patients (45.2%) had "1 or 2 diseases", and eleven (26.2%) had "3 or more diseases". The most frequent diseases were: hypertension, gastric and cardiovascular disorders. Three patients (7.1%) were smokers. Twenty-one subjects (50%) were using removable dentures. All subjects that comprised the study sample were classified into categories "with subjective symptoms" of lichen planus (n = 39, 92.9%), such as burning sensation or pain, or "without subjective symptoms" (n = 3, 7.1%). The time of duration of subjective symptoms varied from 2 to 216 months, while the mean duration was 43 months. Extra-oral manifestations were diagnosed in 30 subjects (71.4%). Skin involvement was present in 15 cases (35.7%). In 4 subjects (9.6%), the involvement of other mucosa was observed. Skin and other mucosa lesions were controlled by the dermatologist and the gynecologist. Lesions on the nail were observed in 27 patients (64.3%). The mean site score according to the Escudier, et al.^6^ (2007) methodology was 4.67(±3.17) points and varied from 1 to 13 points, while the activity mean score was 5.30 (±5.15) points and varied from 1 to 24 points. The mean pain sensation estimated by using the subjective VAS scale was 4.6 (±1.91) on a 10-point scale.

There was a significant positive correlation between the VAS score and severity of OLP (r=0.32; p=0.04) and also a positive non-significant correlation between the VAS score and activity lesions score (r=0.26; p=0.09).

The psychological and psychopathological characteristics of the study group are presented in Table 1.

**Table 1:** The psychological and psychopathological characteristics of the study group

Psychometric tools		N	Mean	Min	Max	SD
The Perceived Stress Scale	PSS Raw score	42	18.6	8.0	29.0	5.2
	PSS Sten Score	42	6.12	3.00	9.00	1.59
The Hospital Anxiety and Depression Scale	HADS Anxiety Score	42	7.62	0.00	18.00	4.08
	HADS Depression Score	42	5.05	0.00	15.00	3.92
The Pain Coping Strategies Questionnaire	CSQ Diverting Attention	39	12.5	0.0	28.0	8.4
	CSQ Reinterpreting Pain Sensations	39	7.95	0.00	24.00	7.43
	CSQ Catastrophizing	39	8.15	0.00	36.00	7.38
	CSQ Ignoring Sensations	39	11.8	0.0	24.0	7.8
	CSQ Praying and Hoping	39	17.2	0.0	33.0	9.1
	CSQ Coping Self-statements	39	20.0	0.0	36.0	9.4
	CSQ Increased Behavioral Activities	39	14.7	0.0	32	9.1
	CSQ Pain Controll	39	3.54	0.00	6	1.25
	CSQ Pain Behaviour	39	3.10	0.00	6	1.19
The Psychological General Well-Being Index	PGWBI Anxiety	41	15.2	5.0	25	5.0
	PGWBI Depression	41	10.2	1.0	15	2.8
	PGWBI Positive Well-Being	41	9.76	2.00	15	2.69
	PGWBI Self-control	41	10.6	3.0	15	2.9
	PGWBI General Health	41	7.73	3.00	13	2.53
	PGWBI Vitality	41	11.20	2.0	20	3.4
	PGWBI Total General Well-Being	41	63.1	0.0	98	19.3

### The correlation between sex, age, general health and quality of life

The correlation analysis that examined the relationship between the patient's age and quality of life revealed a significantly worse quality of life in older patients on the subscale of positive well-being (r=-0.32; p=0.04). The patient's sex had no effect on the general well-being, perceived stress or coping strategies for pain.

The higher number of general diseases in the study sample was related to a significantly poorer quality of life in terms of depression (r=-0.40; p=0.01), positive well-being (r=-0.43; p=0.005), self-control (r=-0.48; p=0.002), anxiety (r=-0.37; p=0.02), vitality (r=-0.34; p=0.03) and total general well-being (r=-0.37; p = 0.01). Moreover, a greater number of general diseases was correlated with a higher level of perceived stress (r=0.41; p=0.006) and with a greater tendency to use catastrophizing (r=0.32; p=0.04) as the coping strategy for dealing with pain. No correlation between the number of general diseases and other coping strategies for pain was observed.

### The relationship between the severity of lichen planus and psychological factors

The correlation between the severity of lichen planus and psychological factors are shown in Table 2.

**Table 2:** The correlation between the duration of the subjective symptoms, the presence of extra-oral manifestations, pain and psychological factors. r=correlation coefficient; * p=0.05 was considered statistically significant

	Duration of subjective symptoms	Presence of extra-oral manifestations	Pain (VAS)
PSS Raw Score	r=0.34	r=0.21	r=0.22
	p=0.03*	p=0.2	p=0.2
PSS Sten Score	r=0.33	r=0.17	r=0.21
	p=0.03*	p=0.3	p=0.2
CSQ Diverting Attention	r=-0.05	r=0.22	r=-0.16
	p=0.7	p=0.2	p=0.3
CSQ Reinterpreting Pain Sensations	r=0.10	r=0.20	r=0.08
	p=0.5	p=0.2	p=0.6
CSQ Catastrophizing	r=0.29	r=0.21	r=0.22
	p=0.08	p=0.2	p=-0.2
CSQ Ignoring Sensations	r=-0.9	r=0.37	r=0.00
	p=0.581	p=0.02*	p=1
CSQ Praying and Hoping	r=0.00	r=0.24	r=0.07
	p=1	p=0.1	p=0.7
CSQ Coping Self-statements	r=-0.06	r=0.25	r=-0.21
	p=0.7	p=0.1	p=0.2
CSQ Increased Behavioral Activities	r=-0.06	r=0.42	r=-0.23
	p=0,7	p=0.008*	p=0.2
CSQ Pain Controll	r=-0.04	r=0.08	r=-0.15
	p=0.8	p=0.6	p=0.4
CSQ Pain Behaviour	r=0.02	r=0.10	r=-0.25
	p=0.9	p=0.6	p=0.1
PGWBI Anxiety	r=-0.23	r=-0.09	r=-0.22
	p=0.2	p=0.6	p=0.2
PGWBI Depression	r=-0.28	r=-0.11	r=-0.30
	p=0.01*	p=0.5	p=0.06
PGWBI Positive Well-Being	r=-0.23	r=-0.06	r=-0.16
	p=0.2	p=0.7	p=0.3
PGWBI Self-control	r=-0.49	r=-0.8	r=-0.19
	p=0.001*	p=0.6	p=0.2
PGWBI General Health	r=-0.18	r=-0.9	r=-0.31
	p=0.3	p=0.6	p=0.05*
PGWBI Vitality	r=-0.17	r=0.00	r=-0.17
	p=0.3	p=1	p=0.3
PGWBI Total General Well-Being	r=-0.17	r=-0.12	r=-0.19
	p=0.3	p=0.4	p=0.2

A longer duration of the subjective symptoms was related to a significantly poorer quality of life in terms of self-control (r=-0.49; p=0.001) and to a higher level of perceived stress (r=0.33; p=0.03). Additionally, a negative statistical correlation between the duration of the subjective symptoms and depression (r=-0.28; p=0.08) was observed. The depression subscale is a so-called reverse scale, meaning that the higher the values on this scale, the lower the level of depression. In turn, this means that the longer the duration of subjective symptoms, the higher the level of depression. Moreover, no correlation between the time of duration of subjective symptoms and different coping strategies was found.

The presence of extra-oral manifestations significantly correlated with increased pain coping strategies such as behavioral activities (r=0.42; p=0.008) and ignoring sensations (r=0.37; p=0.02).

The severity of pain measured with the use of the VAS scale was related to a worse quality of life in the general health subscale (r=-0.31; p=0.05).

There was no significant relationship between the prevalence of psychological factors and psychopathological symptoms in terms of extension, severity and activity of the oral lichen planus lesions and presence of removable dentures.

### Correlation between perceived stress and other psychological/psychopathological factors

A correlation analysis showed strong negative correlation between the level of perceived stress and the quality of life, in which a higher level of perceived stress correlated with lower scores in quality of life domains, such as: positive well-being (p=0.000), self-control (p=0.000), general health (p=0.000), vitality (p=0.000), total general well-being (0.000) as well as anxiety (p=0.000) and depression (p=0.000) – both are so-called reverse scales. Additionally, more frequent use of catastrophizing (p=0.03) as a coping strategy for pain was found in patients with higher stress perception. There was a relationship between higher level of perceived stress and anxiety (p=0.001) and depression (p=0.000) in the HADS scale. The correlation between perceived stress, anxiety and depression (HADS) and the quality of life (PBWBI) is presented in Table 3.

**Table 3:** Correlation between perceived stress, anxiety and depression, the quality of life and coping strategies for pain. r=correlation coefficient; * p=0.05 was considered statistically significant

Peceived Stress (PSS_10)		
	r	p-value
HADS Anxiety	0.48	0.001*
HADS Depression	0.58	0.000*
CSQ Diverting Attention	-0.03	0.9
CSQ Reinterpreting Pain Sensations	0.13	0.4
CSQ Catastrophizing	0.35	0.03*
CSQ Ignoring Sensations	-0.10	0.5
CSQ Praying and Hoping	-0.07	0.7
CSQ Coping Self-statements	-0.25	0.1
CSQ Increased Behavioral Activities	-0.13	0.4
CSQ Pain Controll	-0.22	0.2
CSQ Pain Behaviour	-0.19	0.2
PGWBI Anxiety	-0.78	0.000*
PGWBI Depression	-0.69	0.000*
PGWBI Positive Well-Being	-0.66	0.000*
PGWBI Self-control	-0.69	0.000*
PGWBI General Health	-0.61	0.000*
PGWBI Vitality	-0.63	0.000*
PGWBI Total General Well-Being	-0.69	0.000*

### Correlation between Anxiety, Depression (HADS) and other psychological factors

The correlations between anxiety, depression and other psychological factors are illustrated in Table 4. Higher level of anxiety was significantly related to the use of catastrophizing (p=0.001) as a coping strategy for pain and worse quality of life in all measured domains. Likewise, higher level of depression was strongly correlated with worse quality of life in subscales assessing anxiety (p=0.000), positive well-being (p=0.000), self-control (p=0.001), general health (p=0.03), vitality (p=0.000) and general well-being (p=0.000).

**Table 4:** The correlation between anxiety and depression and other psychological factors. r=correlation coefficient; * p=0.05 was considered statistically significant

	r	p-value	r	p-value
CSQ Reinterpreting Pain Sensations	0.06	0.7	-0.02	0.9
CSQ Catastrophizing	0.53	0.001	0.29	0.07
CSQ Ignoring Sensations	-0.13	0.4	-0.17	0.3
CSQ Praying and Hoping	0.01	1	-0.20	0.2
CSQ Coping Self-statements	-0.03	0.8	-0.16	0.3
CSQ Increased Behavioral Activities	-0.09	0.6	-0.24	0.1
CSQ Pain Controll	-0.30	0.07	-0.30	0.07
CSQ Pain Behaviour	-0.14	0.4	-0.22	0.2
CSQ Pain Control	-0.30	0.07	-0.30	0.07
PGWBI Anxiety	-0.65	0.000	-0.70	0.000
PGWBI Depression	-0.71	0.000	-0.72	0.000
PGWBI Positive Well-Being	-0.63	0.000	-0.76	0.000
PGWBI Self-control	-0.60	0.000	-0.51	0.001
PGWBI General Health	-0.40	0.01	-0.35	0.03
PGWBI Vitality	-0.60	0.000	-0.66	0.000
PGWBI Total General Well-Being	-0.57	0.000	-0.58	0.000

## Discussion

Studies on the etiology and pathogenesis of many somatic diseases suggest influence of multiple factors, including psychosocial ones, which have additive action. Based on that, lichen planus, whose etiopathogenesis is not fully known yet, is perceived as a psychosomatic disease^8^ ^,^ ^16^. Currently, an association between different oral mucosal diseases is being studied, including oral lichen planus lesions and psychiatric disorders, which have been studied more frequently over the past several years. Suresh, et al.^21^ (2015) analyzed the prevalence of recurrent aphthous stomatitis (RAS), oral lichen planus and burning mouth syndrome (BMS) and found a higher incidence of those diseases in patients with depression than in the control group. Furthermore, more than 20% of the patients with anxiety in the study group suffered from oral mucosal disorders. In the study on psychosocial factors in patients with OLP, Chaudhary, et al.^2^ (2004) showed significantly higher levels of stress, anxiety and depression in patients with OLP when compared to the healthy group. Those authors did not find significant differences between the OLP group and the positive control group suffering from BMS and different facial pain. In the subsequent investigation, Kalkur and coauthors^11^ (2015), using the Depression Stress Scale (DASS −42), found higher frequency of psychiatric disorders like depression, anxiety and stress in OLP patients in comparison to the control group matched by age and sex. Similar results were published in the study by Valter, et al.^22^ (2013). Authors found significantly higher level of anxiety, depression and stress in OLP patients, but there was no relationship with the acute stage of OLP lesions or their remission. However, Giradi, et al.^7^ (2011) did not find significant association between OLP and symptoms of depression and stress, but there was a tendency for association with anxiety. Thus, conflicting results are present in the literature.

In our study, the possible association between the signs, severity and duration of symptoms of oral lichen planus, as well as the complaints and psychological and psychopathological profile of the patients were assessed. The mean age of OLP patients was 59.9 years, which is in accordance with observations that the disease occurs in adult patients between 40 and 70 years old. Women were 80% of the investigated patients. The predominance of this disease in women has also been noted in other studies^16^. In our study, the VAS score was positively and significantly correlated with the severity of OLP lesions; however, when the VAS score and the activity of lesions were rated, a significant correlation was not found. We can conclude that mainly the level of severity of the disease – and not the number of sites involved – influences the complaints of patients and their feelings of pain or burning sensations.

In a group of 42 participants of the study, 28.6% were considered generally healthy based on anamnesis. Of all patients, 45.2% complained of 2 general disorders, and 26.2%, of 3 or more. Many studies suggest a relationship between somatic health, quality of life and perceived stress. The number of medical comorbidities in the study sample that consist of patients with OLP is often associated with helplessness that may have an impact on the use of catastrophizing as a pain coping strategy; this is conceptualized as a negative cognitive-affective response to anticipated or actual pain. Then, these patients subjectively, negatively assess their own health, which may instigate the development of somatic symptom disorders. As a chronic disease, OLP can persist in the oral cavity for a prolonged time. In the study sample, the longest duration of the disease was 18 years, while the mean duration was 3.6 years. Factors associated with the disease, such as duration of the disease and presence of extra-oral manifestations, significantly affected the mental state of the patients, which is in accordance with earlier studies by Eisen^5^ (2002) and Soto, et al.^20^ (2003). These authors emphasized the correlation between stress and anxiety and exacerbation of lichen planus oral lesions. In this study, there was a relationship between the duration of the disease and the level of perceived stress, as well as the quality of life (especially the self-control domain). It seems that, the longer the patient needs to struggle with the underlying symptoms, the more likely he/she will develop a sense of loss of control. In severe cases, this may lead to acquired helplessness, causing patients to avoid medical treatment. Moreover, 30 out of 42 patients with OLP had concomitant skin, nail and/or mucosal involvement. The presence of other symptoms increases the likelihood of choosing specific cognitive pain coping strategies, such as ignoring sensations and increased behavioral activity. However, these activities – such as visiting friends to stay busy and unfocused on pain – are not focused on the treatment of symptoms or search for help

Considering the psychopathological consequences, the results of our study emphasize the relationship between perceived stress and symptoms of anxiety and depression, as well between perceived stress and the catastrophizing as a cognitive coping strategy in the study sample. Moreover, catastrophizing was found to positively correlate with anxiety. Based on the results of this study, the psychological profile and the psychopathological consequences of OLP in patients may be determined. Patients with OLP are mostly middle-aged women. As the duration of the diseases increases, patients have an increased tendency to use catastrophizing as a pain coping strategy. That, in turn, increases anxiety and depression and decreases the patient quality of life. These findings are in accordance with the findings of other authors, who postulated that OLP may be considered a psychosomatic disorder^16^. Although the main objective of the study was to assess psychopathological and psychological correlates of clinical characteristics in OLP patients, the limitations of this study must be pointed out, as the observational study design and lack of control group.

The results of our study confirmed the previous findings of other authors and suggest a need for additional therapeutic interventions, including psychological or psychiatric services for patients who have stress-induced oral diseases such as OLP^9^. However, based on the obtained evidence, we can conclude that effective treatment of clinical stage of OLP will reduce their experience of pain, subjective discomfort and anxiety. It will eliminate one of the major stressors to which patients are exposed and reduce their chance to develop depressive symptoms and significantly improve their quality of life.
